# *In silico* identification and verification of Tanshinone IIA-related prognostic genes in hepatocellular carcinoma

**DOI:** 10.3389/fimmu.2024.1482914

**Published:** 2024-10-31

**Authors:** Lichao Qian, Zhongchi Xu, Tianjiong Luo, Zhao Gao, Kun Cheng, Xiaolong He, Zhongai Zhang, Shuai Ren, Yinxing Zhu

**Affiliations:** ^1^ Department of Geratology, Nanjing Hospital of Chinese Medicine Affiliated to Nanjing University of Chinese Medicine, Nanjing, Jiangsu, China; ^2^ College of Biotechnology and Pharmaceutical Engineering, Nanjing Tech University, Nanjing, Jiangsu, China; ^3^ Department of General Surgery, The First People’s Hospital of Taian, Taian, Shandong, China; ^4^ Department of Radiology, Affiliated Hospital of Nanjing University of Chinese Medicine (Jiangsu Province Hospital of Chinese Medicine), Nanjing, Jiangsu, China; ^5^ Department of Traditional Chinese Medicine, Taizhou Hospital of Traditional Chinese Medicine, Taizhou, Jiangsu, China

**Keywords:** Tanshinone IIA, hepatocellular carcinoma, network pharmacology, prognostic model, gene signatures

## Abstract

**Background:**

Currently, adequate treatment and prognostic prediction means for Hepatocellular Carcinoma (HCC) haven’t entered into medical vision. Tanshinone IIA (TanIIA) is a natural product, which can be utilized as a potential treatment of HCC due to its high anti-tumor activity. However, the effect on HCC prognosis, as well as the potential targets and molecular mechanism of TanIIA still remain ambiguous. Herein, we investigated them via network pharmacology, explored TanIIA-related prognostic genes by machine learning methods, and verified using molecular docking and cell experiments.

**Methods:**

Potential TanIIA-targeted genes and HCC-related genes were obtained from the corresponding database. The Protein-Protein Interaction (PPI) network and enrichment analyses of the intersection targets were conducted. Furthermore, a TanIIA-related prognostic model was built and verified. We attempted to explore the expression of the TanIIA-related prognostic genes and evaluate its chemotherapeutic sensitivities and the immune infiltrations. Followed by exploration of anti-tumor activity on the human HCC cells Hep3B and HepG2 cell lines *in vitro* (CCK-8, flow cytometry and transwell assay), the docking molecular was performed. Ultimately, the corresponding protein expressions were determined by western blotting.

**Results:**

A total of 64 intersecting targets were collected. Similarly, GO/KEGG enrichment analysis showed that TanIIA can inhibit HCC by affecting multiple pathways, especially the MAPK signaling pathway. A five-gene signature related to TanIIA was constructed on account of Least Absolute Shrinkage and Selection Operator (LASSO) Cox regression model. Among five genes, ALB, ESR1 and SRC tend to be core genes because of probable status as potential targets for sorafenib. Molecular docking results demonstrated the potential for active interaction between the core genes relevant proteins and TanIIA. Studies *in vitro* had shown that TanIIA regulated the expressions of Bcl-2, Bax and MMP9 in HCC cells, inhibiting their growth, inducing apoptosis and preventing cell invasion. Additionally, we are able to detect an up-regulated trend in the expression of ALB and ESR1, while a down-regulated in the expression of SRC by TanIIA.

**Conclusion:**

Regulating the expression of TanIIA-related gene signatures (ALB, SRC and ESR1), and inhibiting the SRC/MAPK/ERK signaling axis might potentially contribute to the TanIIA treatment of HCC. And the three gene signatures could be identified for predicting the prognosis of HCC, which may provide novel biomarkers for HCC treatment.

## 1 Introduction

Hepatocellular carcinoma (HCC) accounts for about 85%-90% of all kinds of primary liver cancers (1), which seriously endangers human health. With regard to the treatment of HCC, surgical resection is still the only kind of radical cure. However, only one-third of patients with HCC at early stage can receive the treatment. Unfortunately, most of the patients are in the middle and advanced stages when clinically diagnosed. They can only receive non-radical treatment, such as transcatheter arterial chemoembolization (TACE), drug chemotherapy, molecular targeted therapy and immunotherapy (2). Even so, the 5- year survival rate of these patients is still lower than 12.5% (3). Hence, the patients of HCC, especially advanced ones, are urgently requiring more treatment options.

Traditional Chinese medicine (TCM) has been widely accepted as a complementary therapy for tumors (4). In multiple studies, Salvia miltiorrhiza has been proved to have anti-tumor activity and can confer sensitivity to various drug-resistant tumors (5). In recent preclinical investigations, Salvia miltiorrhiza has demonstrated a diverse pharmacological profile, rendering it a potential therapeutic agent for a spectrum of liver pathologies. This traditional medicinal herb exerts hepatoprotective effects by shielding the liver from the detrimental impacts of hepatotoxins, alleviating hepatic oxidative stress, ameliorating steatosis, and diminishing inflammatory, fibrotic, and neoplastic processes within the liver (6). Furthermore, both Salvia miltiorrhiza and its derived extracts have been shown to exert anti-proliferative activities against HCC cells, underscoring their chemopreventive potential (7, 8).

The main chemical components of Salvia miltiorrhiza, including tanshinones I, IIA, IIB, V, VI, cryptotanshinone, etc., have a wide range of anti-tumor biological activities. They can inhibit the proliferation and induce apoptosis of the vast majority of tumor cells (9, 10).

HCC is a complex disease influenced by a multitude of genetic and epigenetic factors that regulate its initiation and progression (11, 12). Among the various compounds found in Salvia miltiorrhiza, TanIIA has been identified as one of the most promising due to its high number of potential anti-tumor targets, particularly against HCC (13–15). This compound has been recognized for its multifaceted therapeutic characteristics, including the ability to interact with multiple targets and pathways associated with HCC development and progression. Despite its recognized potential, the precise mechanism by which TanIIA contributes to the treatment of HCC and its impact on patient prognosis remains to be fully understood. The advent of network pharmacology offers a new approach to elucidate the complex interactions between TanIIA and the biological systems it engages, including the intricate network of genes and pathways implicated in HCC. This holistic perspective may provide insights into the multi-targeted action of TanIIA and its potential for improving outcomes in HCC treatment.

Network pharmacology is a novel technique, which integrates traditional pharmacology, molecular biology, data mining, biological information and network analysis. And it can change the current situation of single target research. Through network construction, the association between “drug-multitargets-multipathways-disease” can be visualized and comprehensively studied from a holistic perspective. And the detailed mechanism of drugs can be systematically predicted, which coincides with the holism, multi-components, multi-targets and multi-pathways of TCM (16). In this study, we investigated the network pharmacology of TanIIA in the treatment of HCC.

**Figure 1:**
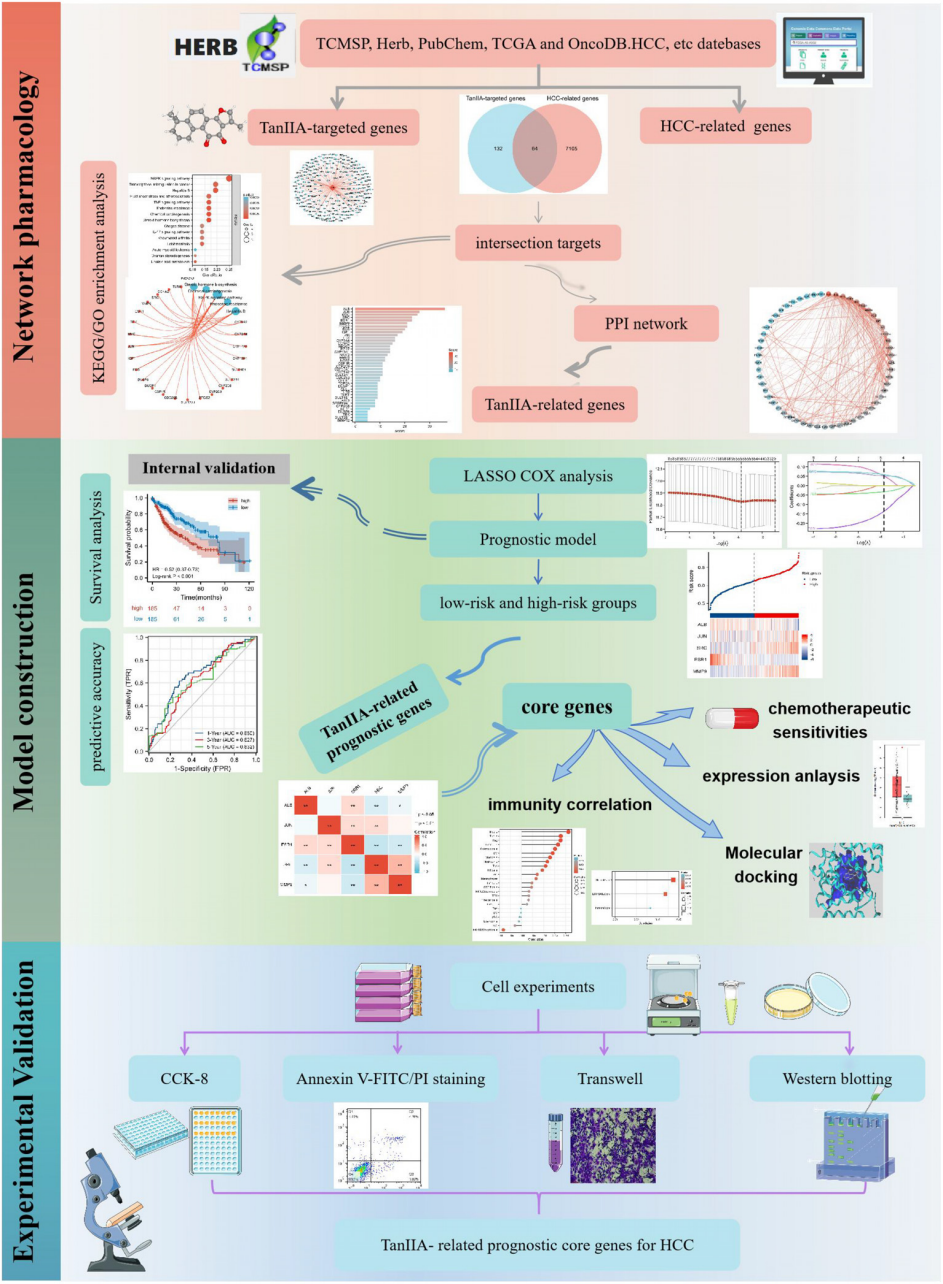
The whole framework based on the methods of network pharmacology, machine learning, and experimental verification.

Network pharmacology was adopted to identify the putative targets of TanIIA in the treatment of HCC in this study, and the potential mechanisms involved in the treatment of HCC were further explored. Then the prognostic value of targets of TanIIA was explored via Cox expression analysis, and a prognostic model was established based on these TanIIA-related genes of prognostic value. Subsequently, the relationships between the TanIIA-related prognostic genes and tumor immune microenvironments were investigated, and the drug sensitivity of these genes were evaluated as well. Finally, the findings of network pharmacology and prognostic model were validated *in-vitro* experiments including cell assays, transwell and Western blotting. It is concluded that TanIIA has a good therapeutic effect in HCC therapy via muti-targets and muti-pathways, and the TanIIA-related genes are potential targets for the HCC prognosis and treatment. The whole framework of this study is shown in **Figure 1**.

## 2 Materials and methods

### 2.1 Network pharmacology

#### 2.1.1 Collection of potential TanIIA-targeted genes

The TanIIA-targeted genes were obtained from TCMSP (https://tcmsp-e.com/) (17), Herb (http://herb.ac.cn/) (18), PubChem (http://pubchem.ncbi.nlm.nih.gov/), STITCH databases (http://stitch.embl.de/) (19) and HIT (http://hit2.badd-cao.net) (20) by searching “Tanshinone IIA”. In addition, the SMILE format of TanIIA was copied to SwissTargetPrediction database (http://www.swisstargetprediction.ch/) (21) to predict the relevant targets of Probability > 0.1. Moreover, the TanIIA-targets and gene names were screened by Normfit ≥ 0.3 and Zscore≥ 0.5 from PharmMapper database (http://lilab.ecust.edu.cn/pharmmapper/ ) (22) via uploading the 3D structure of TanIIA. The final targets were used to construct a TanIIA-TanIIA target network.

#### 2.1.2 Collection of HCC-related genes

HCC-related genes were obtained from the GeneCards database (https://www.genecards.org), OMIM (https://www.omim.org) and Drugbank databases (https://www.drugbank.com/) using “hepatocellular carcinoma” or “hepatoma” as keywords. Additional related genes were collected from the OncoDB.HCC database(http://oncodb.hcc.ibms.sinica.edu.tw/index.htm) (23).

#### 2.1.3 Construction of TanIIA-related genes network

To obtain the potential targets for TanIIA treatment of HCC, the HCC-related genes were crossed with the TanIIA-targets. Then the intersection targets were introduced into the STRING database (https://www.string-db.org/). The targets with confidence score > 0.4 were employed to carry out the protein-protein interaction (PPI) network visualization and analysis. The degree of each protein was calculated by Cytoscape3.8.0 software plug-in, and the proteins with a degree value > 5 were screened as TanIIA-related genes.

#### 2.1.4 Enrichment analysis of TanIIA-related genes

The R package “clusterProfiler” was employed to perform the functional enrichment analysis of the intersection targets, including gene ontology (GO) and Kyoto Encyclopedia of Genes and Genomes (KEGG) analyses. Terms and pathways with *P* values < 0.05 were screened, and the top 15 enriched GO terms and KEGG pathways were visualized via “ggplot2” package (24).

### 2.2 Model construction and identification

#### 2.2.1 Model construction

The Univariate Cox analysis conducted via the “survival” R package was employed to find out the TanIIA-related prognostic genes (*p*< 0.05). And the efficient prognostic model was established by LASSO analysis with “glmnet” R package. And the overall survival (OS) was chosen as the response variable. Meanwhile, the minimal penalty term (λ) with ten-fold cross-validation was unitized. And the risk score of HCC patients from the TCGA cohort was calculated according to the formula: risk score=sum (gene expression level × corresponding coefficient). Thereafter, the patients were classified into low-risk and high-risk groups on basis of the median risk score. The “survminer” and “timeROC” package were used to conduct survival analysis and predictive accuracy.

#### 2.2.2 Identification of the core genes and exploration of their characteristics

The GEPIA2 database (http://gepia2.cancer-pku.cn/) (25) was employed to examine the expression of the prognostic genes in HCC. The Spearman’s correlation analysis and “pheatmap” package were performed to analyze and display the correlation between prognostic genes. The genes which had statistically significant correlation with the candidates were considered as the core genes.

The chemotherapeutic sensitivities to sorafenib between difference expression of the prognostic genes were predicted by the R package “pRRophetic”. In accordance with the Genomics of Drug Sensitivity in Cancer (GDSC) database (https://www.cancerrxgene.org/), the IC_50_ estimates were obtained via ridge regression.

Single-sample gene set enrichment analysis (ssGSEA) was applied to explore the relationship between the expression of core genes and immune cells, and the infiltrations of immune cells were quantified utilizing the GSVA package. In addition, the “ESTIMATE” package was employed to compute the immune, ESTIMATE, and stromal scores. Then, the correlation between the expression levels of core genes and immunity were analyzed by the Spearman’s correlation.

#### 2.2.3 Molecular docking verification

Molecular docking models for the identified core genes and TanIIA were constructed to evaluate prognostic targets. The PDB formats of the above compounds were retrieved from RCSB (http://www.rcsb.org) database. 3D chemical structural formulas were made via ChemBioDraw 3D. Then they were modified by the SYBYL-X (version 2.0, TRIPOS Inc.) software, including ligands and water removal, amino acid patching and optimization, hydrogen addition and active pocket construction. Moreover, SYBYL-X software was used for docking and evaluating the docking scores, which represents of binding affinities between TanIIA and core genes. Finally, the results were also visualized in SYBYL-X software. In general, the CSCORE >4.0 indicates good binding activity with the molecules (26).

### 2.3 Experimental validation

#### 2.3.1 Cell culture and TanIIA administration

The two hepatocellular carcinoma cell lines, HepG2 and Hep3B, were purchased from Procell Life Science & Technology Co. Ltd. (Wuhan, China) and cultured in DMEM medium with 1% streptomycin and penicillin (KeyGEN biotech, Nanjing, China) supplemented with 10% FBS (GIBCO, NY, US). The cells were incubated at 37°C with 5% CO_2_.

TanIIA (purity ≥98%) was purchased from Yuanye Biotech (Shanghai, China) and dissolved in DMSO at concentrations of 5 m. The TanIIA solution was stored at -20°C.

#### 2.3.2 Cell counting Kit-8 assay

Cell proliferation was determined by the CCK-8 assay (Beyotime Institute of Biotechnology, Shanghai, China). HepG2 and Hep3B cells were seeded in 96-well plates at a density of 3 × 10 (4)/well. When adhered to the wall, the cells were intervened with various concentrations of TanIIA at 0, 20, 40, 60, 80, 100 and 120 μmol for 24h. The control (non-intervened) cells (0 μmol) were in DMEM containing 0.3% DMSO. According to the manufacturer’s protocol, CCK-8 reagent (10 µL/well) was added to the cells, and cells were cultured for 30min at 37°C. The absorbance was measured by a microplate spectrophotometer (Thermo Scientific, USA) at 450 nm. The cell viability rates (%) of the two cells were calculated as experimental absorbance value/control absorbance value × 100. Moreover, The IC_50_ were evaluated via GraphPad Prism 8.0 software.

#### 2.3.3 Flow cytometry assay

The detection of TanIIA-induced apoptosis was determined by Flow cytometry assay (FCM). Hep3B and HepG2 cells were respectively implanted in 6-well plates at a density of 1 × 10 (5) cells/well overnight. Then, the cells were intervened with a series of different concentrations of TanIIA solution for 24 h. After harvested, the above cells were washed and resuspended gently with PBS. Whereafter, 5 μL Annexin V-FITC and 5 μL PI staining solution were added into cells. They were mixed gently and incubated in the dark at room temperature for 15 min. The cell apoptosis was then detected by FCM (BD Biosciences, USA).

#### 2.3.4 Transwell assay

Transwell assay was employed to measure the invasive ability. Hep3B and HepG2 cells were intervened with TanIIA for 24 h. The inserts (8-mm pore size; corning, inc.) in the 24-well plate were coated with diluted Matrigel (BD Biosciences, USA) initially. The intervened cells were trypsinized and resuspended in serum-free medium. Subsequently, 200μl cells (1×10 (5)) were transferred in the upper chambers, and the 24-well plate was filled with 500 μL 10% FBS-containing complete medium. Following 24 h of reseeding, the cells were subjected to 30min 4% paraformaldehyde fixation and 20min 0.1% crystal violet staining. After washing with PBS several times, the cells attached to the upper chamber were removed gently and the inferior surface were visualized under a ×10 microscope. Finally, the cells were quantified utilizing ImageJ software.

#### 2.3.5 Western blotting

The TanIIA-intervened cells were lysed in RIPA buffer (Vazyme Biotech, China). Afterward, 30 µg proteins were isolated by 10% SDS-PAGE and transferred to PVDF membranes (0.45μm). Afterwards, the membranes were blocked in 1-hour 5% non-fat milk at ambient temperature. Subsequently, these blots were incubated overnight at 4°C with specific primary rabbit antibodies against ALB (Proteintech, China, diluted 1:1000), SRC (Proteintech, China, diluted 1:300), ESR1 (Proteintech, China, diluted 1:500), BAX (Cell Signaling Technology (CST), USA, diluted 1:1000), BCL-2 (CST, USA, diluted 1:1000), GAPDH (Proteintech, China, diluted 1:2000). The next day, the membranes were incubated with goat anti-rabbit secondary antibody (Sangon Biotech, China, diluted 1:5000) for 1 hour. In the end, the chemiluminescence image analysis system (Tanon, Shanghai, China) was utilized for protein detection and visualization. The proteins were quantified by ImageJ software, and the relative levels were normalized by comparison to GAPDH.

### 2.4 Statistical analysis

All data analyses were accomplished with R (version 3.6.1) software and Graphpad Prism 8.0. The former and its related packages were applied to conduct Network pharmacology and bioinformatics analyses. Differences between experimental groups were analyzed via Student’s t-test, one-way analysis of variance, and chi square test. All results were exhibited as mean ± SD, and *p< 0.05, **p< 0.01 were regarded as statistically significant.

## 3 Results

### 3.1 Network pharmacology

#### 3.1.1 Pharmacokinetic properties and targeted genes of TanIIA

**Figure 2:**
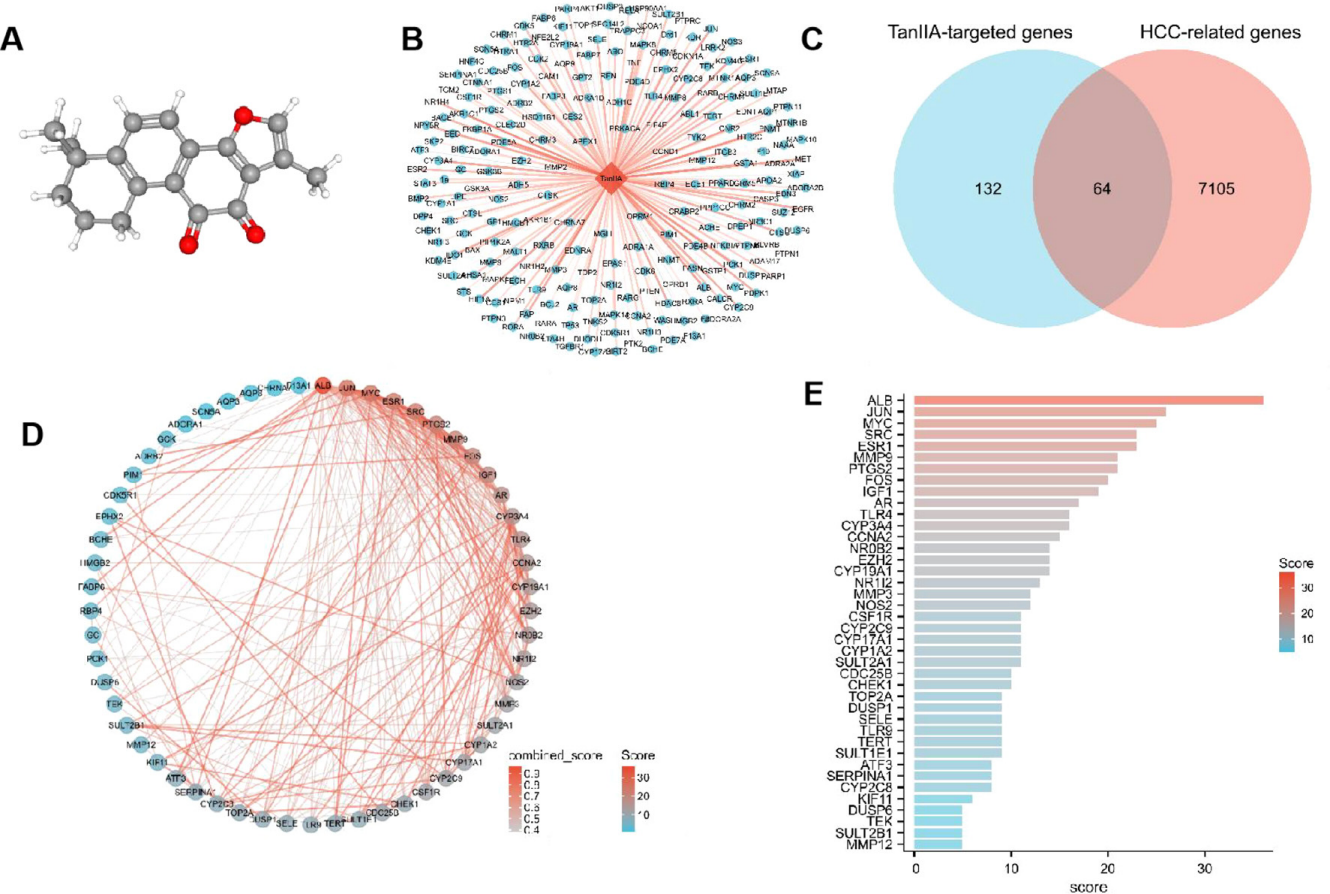
Network pharmacology results. **(A)** 3D chemical structure of TanIIA (PubChem CID, 164676). Its molecular formula is C19H18O3. **(B)** TanIIA-TanIIA target network (The orange diamond indicating TanIIA, and blue circles indicating targets). **(C)** Venn diagram of potential TanIIA-targeted genes and HCC-related genes. **(D)** PPI network of TanIIA-related genes. **(E)** Degree value of interaction network.

The 3D structure of TanIIA is shown in **Figure 2A**. Its pharmacological and molecular properties, obtained from TCMSP, are shown in **Supplementary Table 1.** The number of targets searched from 7 databases was as follows: there were 40 in TCMSP database, 51 in Herb database, 32 in HIT database, 18 in PubChem database, 79 in PharmMapper database, 57 in SwissTargetPrediction database and 7 in STITCH database. A total of 196 potential TanIIA-targeted genes were collected after deduplication. As shown in **Figure 2B**, the TanIIA-TanIIA target network shows 197 nodes (196 targets and TanIIA), which represent the interaction between TanIIA and its potential therapeutic targets.

#### 3.1.2 Identification of HCC-related genes

The number of related genes screened from the 4 databases were 609 in OncoDBHCC database, 6994 in GeneCards database, 245 in OMIM database and 48 in Drugbank database. After removing duplicates, 7169 genes were found.

#### 3.1.3 Identification of TanIIA-related genes and PPI network

The three sets of data obtained above, including TanIIA-targeted genes, HCC-related genes and differential genes, were screened out to have a total of 64 genes, and the Venn diagram was drawn by R package ggplot2 (**Figure 2C**). These 64 genes were defined as TanIIA-related genes. The TanIIA-related genes were introduced into String to construct a PPI network, and the free nodes were removed, resulting in a total of 57 targets (**Figure 2D**). The degree value was sorted by Cytoscape 3.8, and 40 key target proteins with degree value > 5 were screened out. The bar chart of corresponding degree values of target proteins is shown in **Figure 2E**. The top eight target proteins were ALB, JUN, MYC, SRC, ESR1, MMP9, PTGS2 and FOS.

#### 3.1.4 Enrichment analysis of TanIIA-related genes

**Figure 3:**
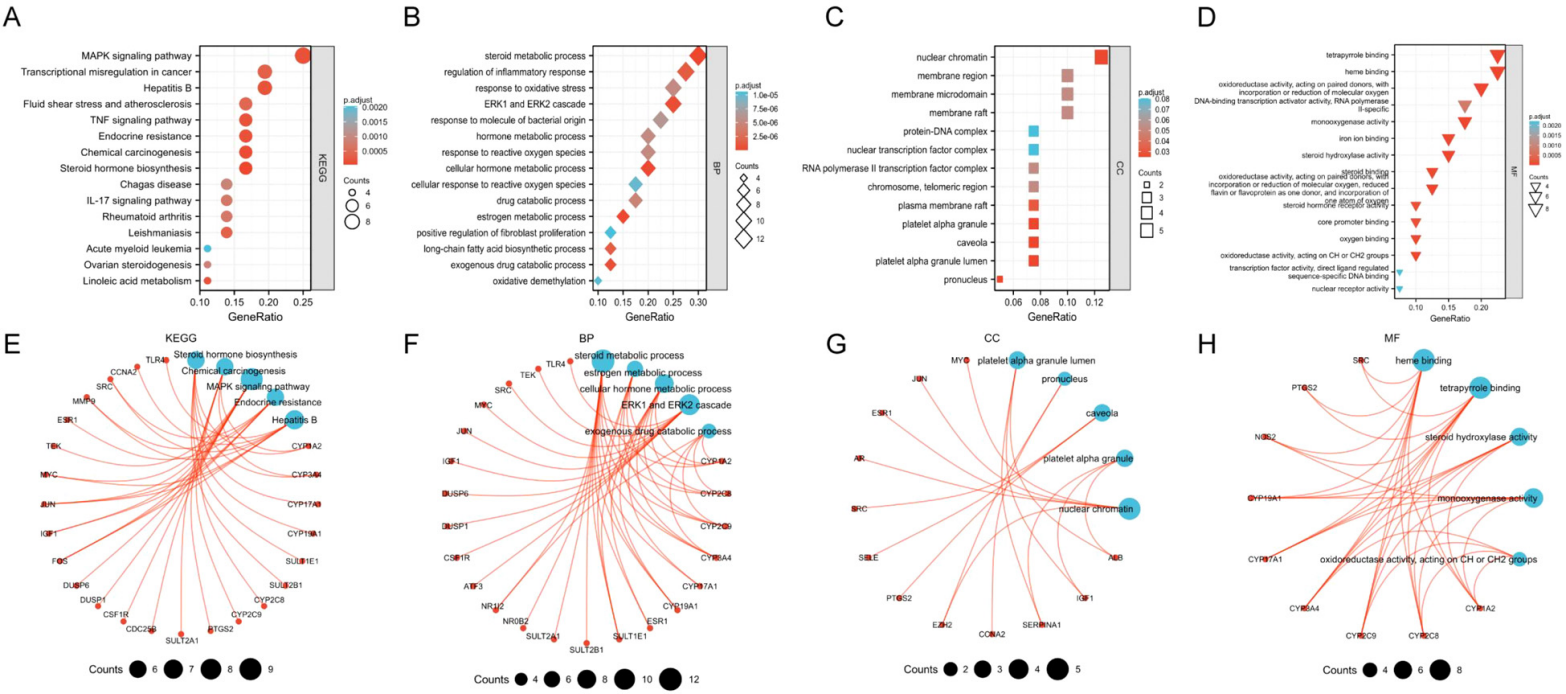
Enriched KEGG pathway and GO terms. The top 15 enriched results in KEGG pathway **(A, E)**, Biological processes **(B, F)**, Cell components **(C, G)**, Molecular functions **(D, H)**.

KEGG/GO enrichment was performed on the 40 key target genes obtained above. On the premise of *p* < 0.05, the top 15 pathways and functional modules are shown in **Figure 3**. From the results of KEGG (**Figure 3A, E**), it is found that the key targets of TanIIA in the treatment of HCC are mainly involved in the MAPK signal pathway, Transcriptional misregulation in cancer, Hepatitis B, TNF signal pathway, endocrine resistance and other signal pathways, indicating that the therapeutic effect of TanIIA on HCC may be realized through the above pathways. GO enrichment results suggested that the biological processes were mainly involved in steroid metabolic process, regulation of inflammatory response, response to oxidative stress, ERK1 and ERK2 cascade and response to molecule of bacterial origin, etc. (**Figure 3B, F**). The cell components were associated with nuclear chromatin, membrane region, membrane microdomain, membrane raft and protein-DNA complex, etc. (**Figure 3C, G**). The molecular functions were related to heme binding, tetrapyrrole binding, oxidoreductase activity, monooxygenase activity, DNA-binding transcription activator activity, etc. (**Figure 3D, H**). Taken together, these indicate that TanIIA mainly affects cell proliferation, apoptosis and migration through its effects on oxidative stress, hormone metabolism, inflammatory immunity, and so on, so as to have therapeutic effects on HCC.

### 3.2 Model construction and identification

#### 3.2.1 Construction and validation of the prognostic model

**Figure 4:**
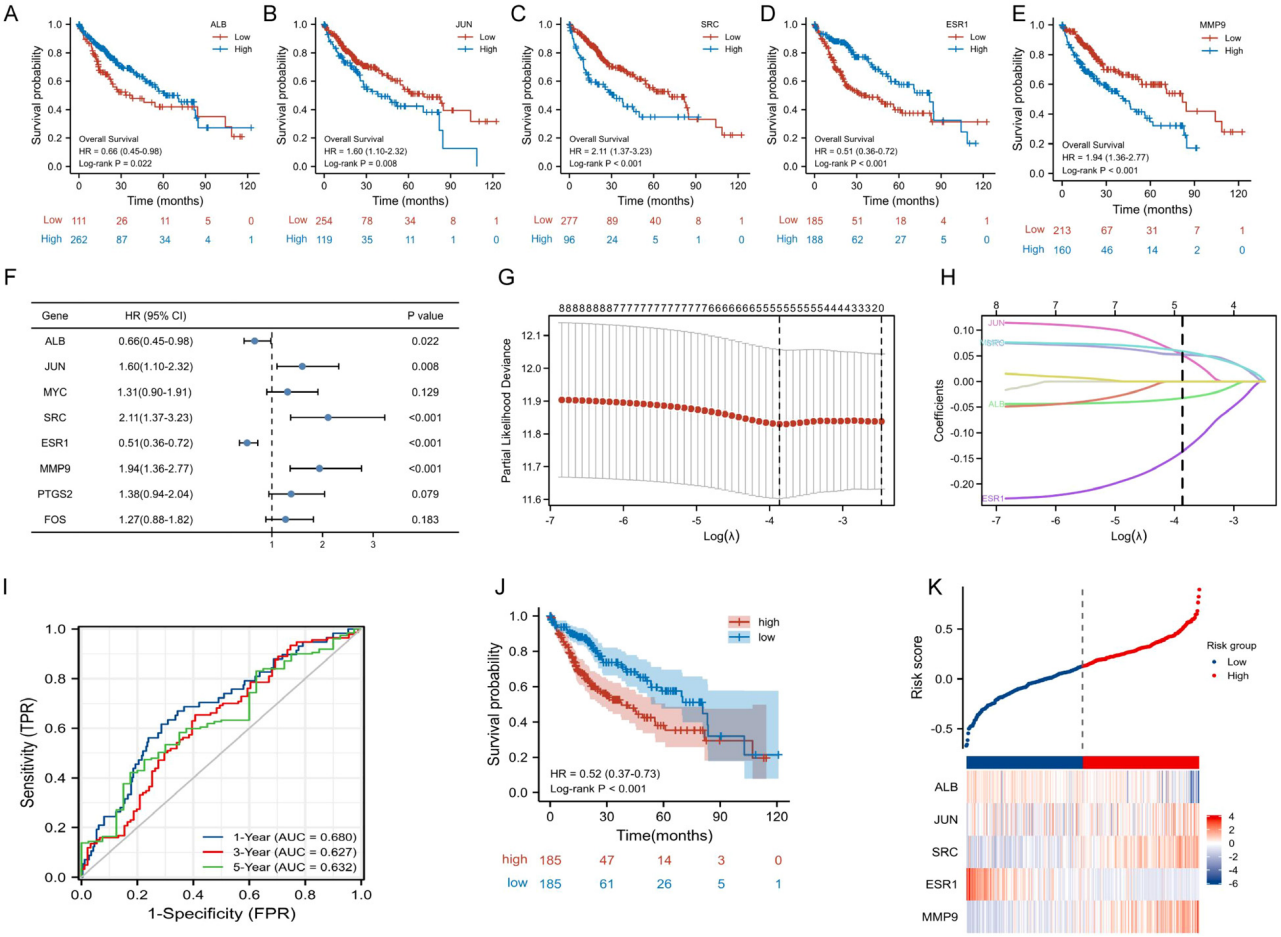
Construction and validation of TanIIA-related prognostic model. **(A-E)**. The prognostic significance of expression level of ALB **(A)**, JUN **(B)**, SRC **(C)** ESR1 **(D)** and MMP9**(E)** in TCGA cohort. **(F)** The forest plot of Hazard ratios for TanIIA-related genes. **(G)** LASSO regression constructed TanIIA-related prognostic model. **(H)** LASSO coefficient profiles. **(I)** The time-dependent ROC curves. **(J)** The K-M curves for the OS between the high- and low-risk groups. **(K)** Detailed risk scores and heatmap of five TanIIA-related genes in TCGA cohort.

To further explore the prognostic value of TanIIA-related genes in HCC, KM analysis and COX in TCGA-LIHC cohort were utilized to assess the prognosis significance of TanIIA-related genes. KM analysis results showed that 5 genes (ALB, JUN, SRC, ESR1 and MMP9) met the criteria of *P* < 0.05, indicating their associations with OS prognosis (**Figure 4A–E**). And among those genes, which were identified to be associated with OS, 3 genes (JUN, SRC and MMP9) were considered as risk factors with HRs >1, while another 2 genes (ALB and ESR1) were protective factors with HR <1 (**Figure 4F**). In addition, LASSO Cox regression analysis was performed to construct a prognosis prediction model. In accordance with the optimum λ value with ten-fold cross-validation, the 5 TanIIA-related gene signatures were selected, and a 5-gene prediction model was established (**Figures 4G, H**).

TCGA-LIHC cohort was used as the training set. Moreover, it was also employed as testing set for internal cross validation. The risk score was derived from the following formula: riskscore = (-0.0324)×ALB expression +(0.0519)×JUN expression +(0.0524)×SRC expression +(-0.1366)×ESR1 expression +(0.0588)×MMP9 expression. Based on the risk scores, HCC patients in TCGA database were stratified into low- and high-risk subgroups. The area under the time-dependent ROC curve (AUC) were 0.680 for 1-year, 0.627 for 3-years, and 0.632 for 5-years (**Figure 4I**). And the KM curve revealed that the low-risk group had better prognostic than the high-risk group. (**Figure 4J**). Besides, it was also analyzed for distribution of selected gene expressions and risk scores in the TCGA cohort (**Figure 4K**).

#### 3.2.2 Identification of the core genes’ characteristics

**Figure 5:**
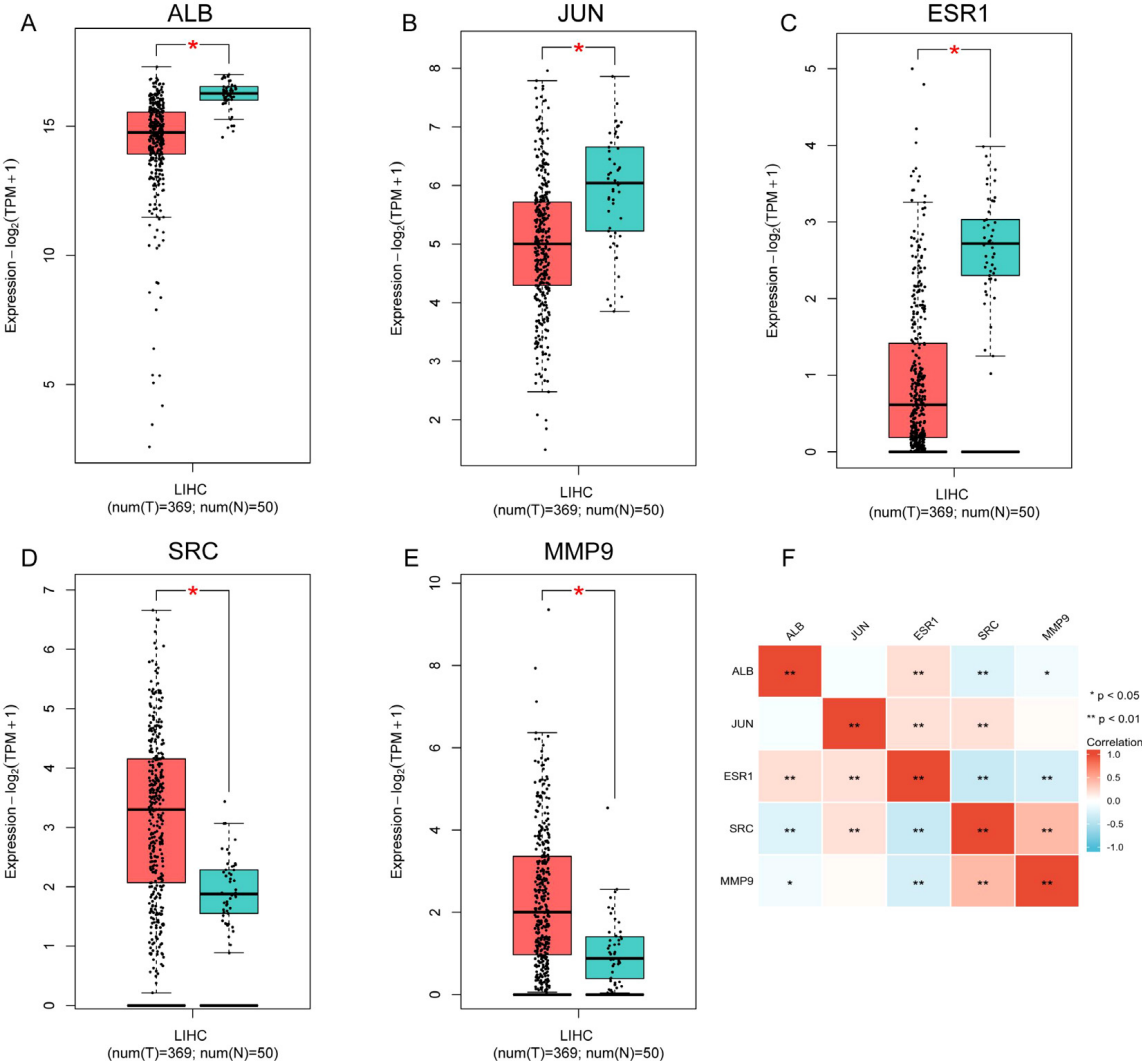
The expression of the five prognostic TanIIA-related genes. **(A–E)** Expression of the five genes in LIHC (Red boxes represent tumor samples and blue boxes represent normal samples). **(F)**. Spearman correlation analysis of the five genes.

The expression of the five prognostic TanIIA-related genes were further analyzed. The analysis results demonstrated that the genes were differentially expressed in HCC and normal samples (**Figures A–E**). And the correlation among the 5 gene expression was shown in **Figure 5F**.

**Figure 6:**
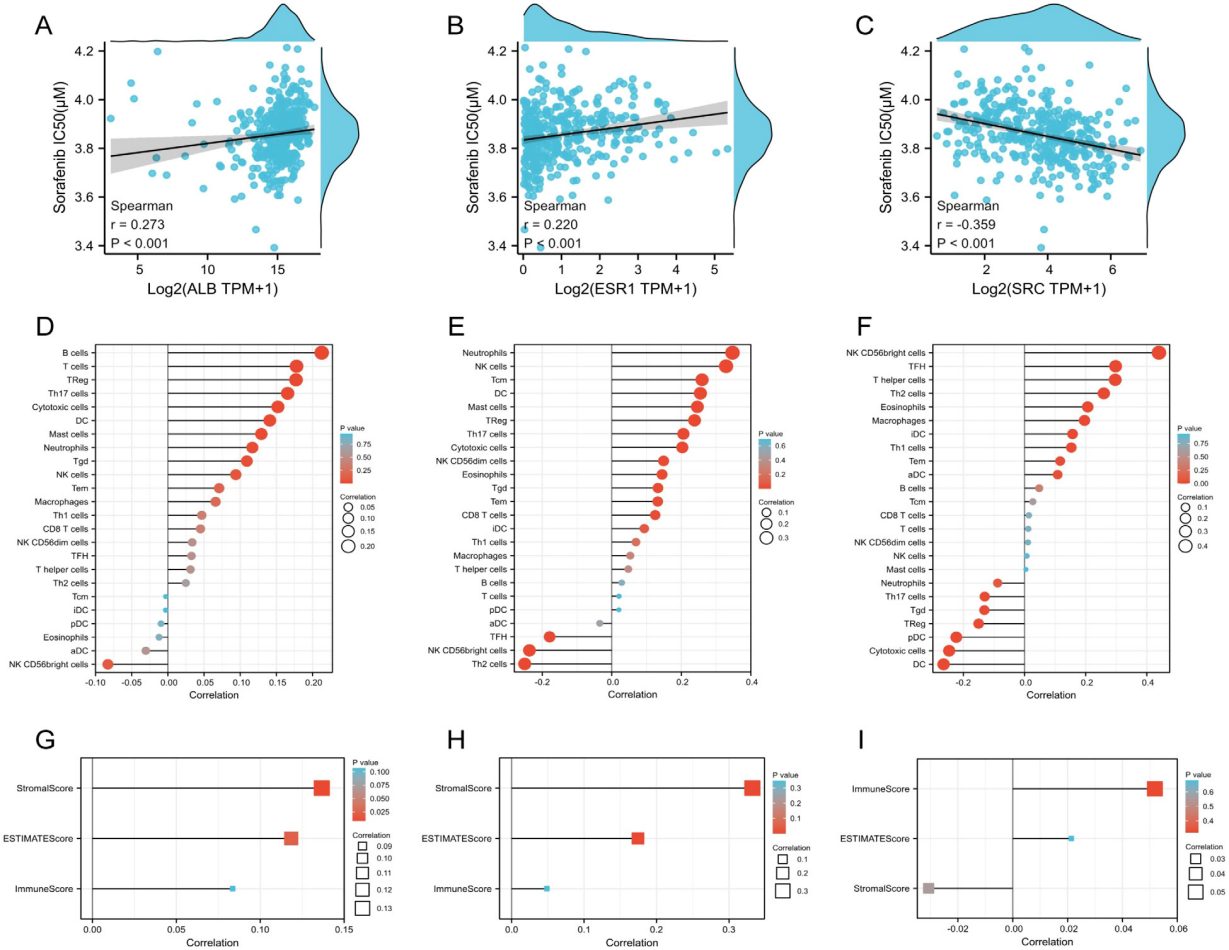
Characteristics of the core genes. **(A–C)** Correlation between Sorafenib sensitivity and the expression of ALB **(A)**, ESR1 **(B)** and SRC **(C)**. **(D–F)** Correlation between immune cell infiltration and the expression of ALB **(D)**, ESR1 **(E)** and SRC **(F)**. **(G–I)** Correlation between immune scores and the expression of ALB **(G)**, ESR1 **(H)** and SRC **(I)**.

Then the relationship between drug sensitivities of Sorafenib and expression levels were evaluated. Among five genes, ALB, ESR1 and SRC was more likely to serve as potential targets for sorafenib. ALB (r=0.273, *p*>0.01, **Figure 6A**) and ESR1 (r=0.220, *p*>0.01, **Figure 6B**) was negatively correlated with sorafenib IC50 value. Meanwhile, SRC was negatively (r=-0.359, *p*>0.01, **Figure 6C**). The results were in accordance with the results of Spearman correlation analysis of the 5 prognostic TanIIA-related genes (**Figure 5F**). Therefore, we chose ALB, ESR1 and SRC as our core genes for further investigation.

The statistical correlation between immunity and expression levels of prognostic TanIIA-related genes were also clarified, including immune cell infiltration and immune scores. It was shown that Neutrophils cells, DC cells, Treg cells, Th17 cells, Tgd cells and Cytotoxic cells, etc. were significantly positively correlated with the expression of ALB and ESR1 (**Figures 6D, E**). While, most of the above positive-correlated immune cells, such as DC cells, Treg cells, Th17 cells and Tgd cells shown a negative correlation with SRC (**Figure 6F**). Moreover, the expression of ALB and ESR1 and the StromalScores and ESTIMATEScores were positively correlated (**Figures 6G, H**), while SRC expression shown no statistically correlation (**Figure 6I**). These results corroborated that the core genes are correlated to the development and progression of HCC.

#### 3.2.3 Molecular docking verification

**Figure 7:**
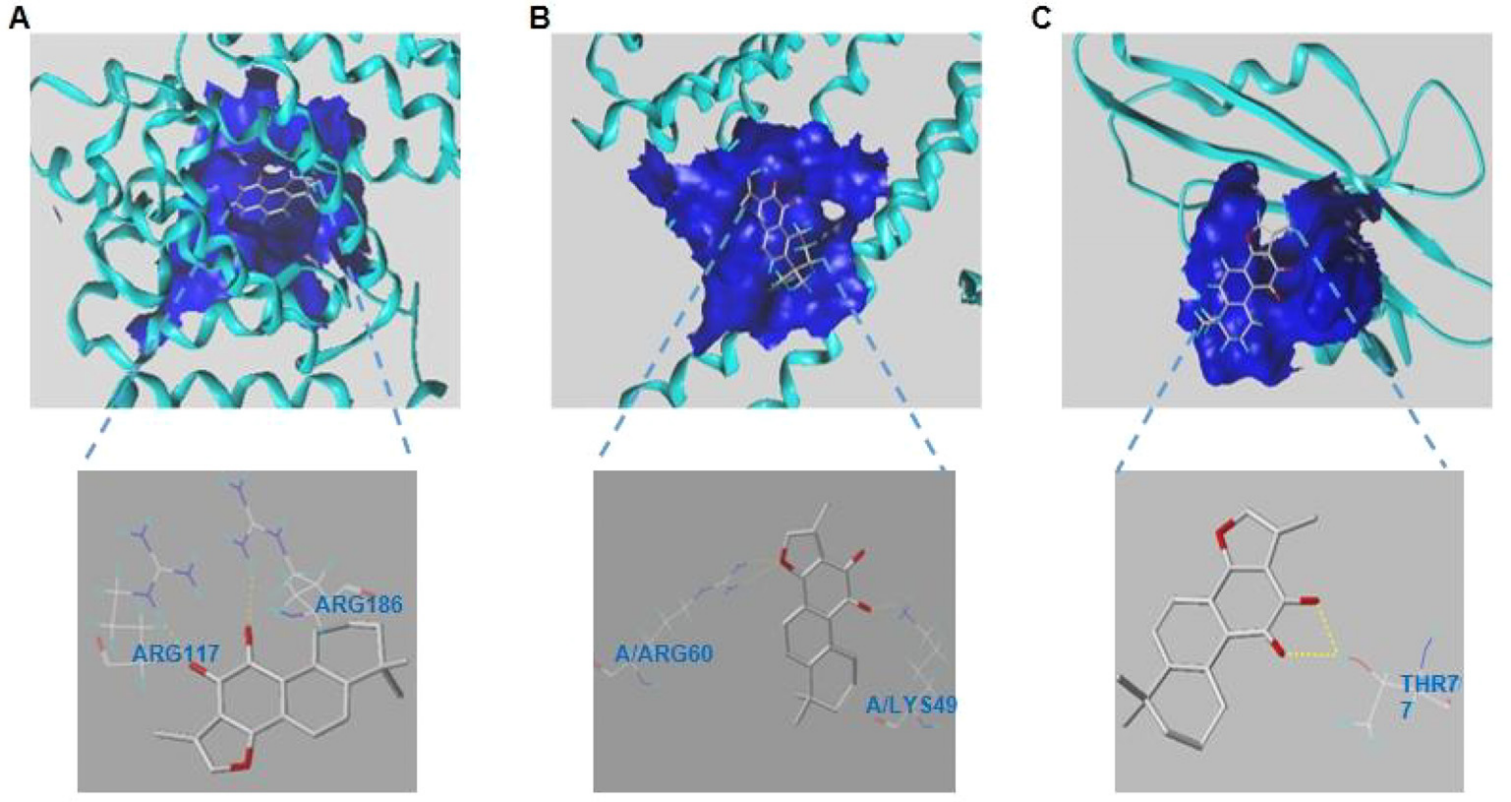
**(A–C)**. Modes of docking between of TanIIA and targets protein molecules. **(A)** ALB. **(B)** ESR1. **(C)** SRC.

Sybyl-X was utilized to locate the binding sites and evaluated the binding score values of the compounds. According to the molecular docking results, TanIIA had well protein affinities with core genes relevant proteins (**Figure 7**). And docking scores were>4, suggesting relatively stable binding activities as well.

### 3.3 Experimental validation

#### 3.3.1 The efficiency of TanIIA on cell proliferation inhibition

**Figure 8:**
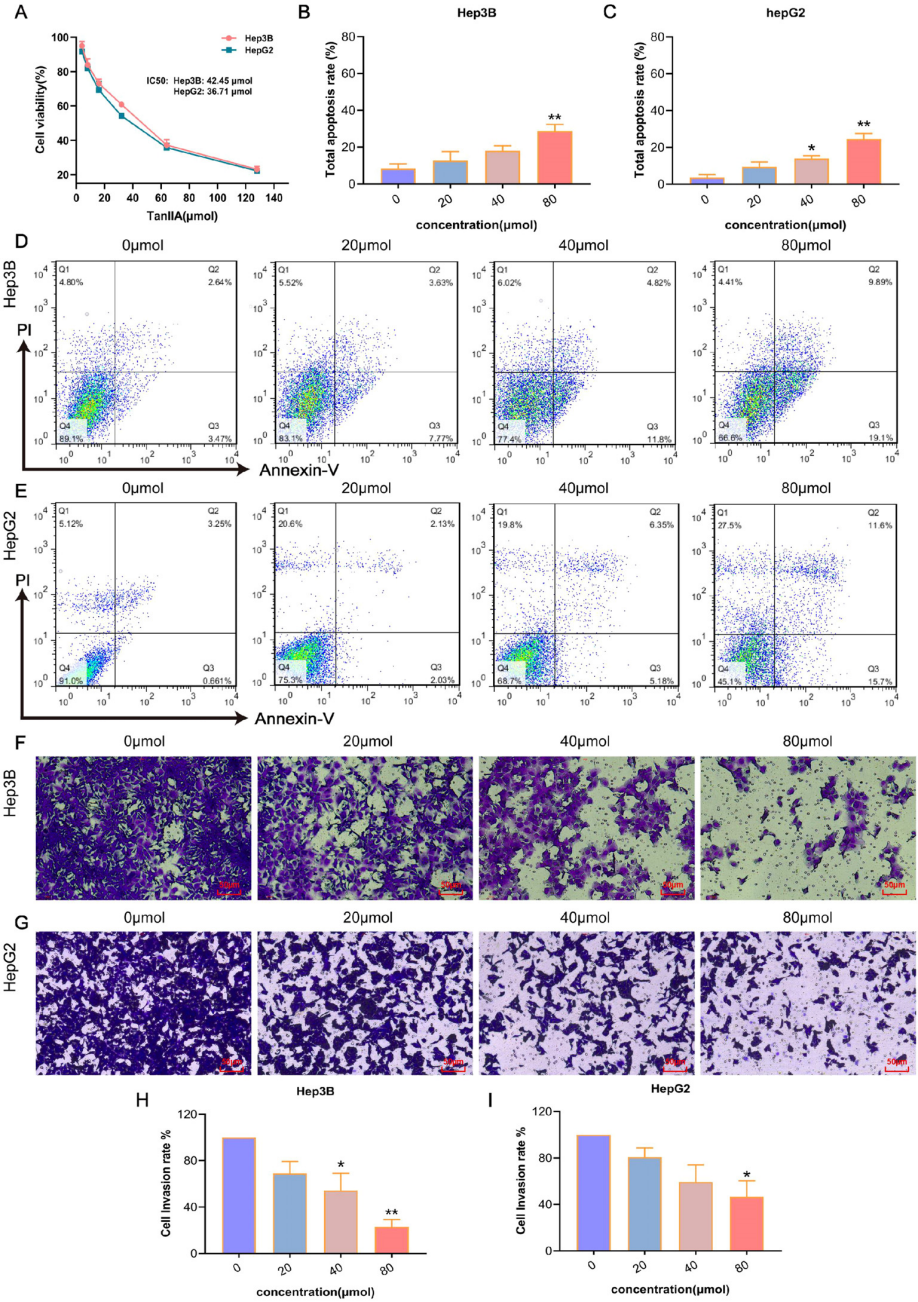
The anticancer efficiency of TanIIA. **(A)** Cell viability of Hep3B and HepG2 cells intervened with DMSO or different dose of TanIIA. B-C. Total apoptosis rates of Hep3B **(B)** and HepG2 **(C)** intervened with various concentrations of TanIIA. D-E. Apoptosis distribution of Hep3B **(D)** and HepG2 **(E)** intervened with 0 μmol **(A)**, 20 μmol **(B)**, 40 μmol **(C)**, 80 μmol **(D)** of TanIIA. F-G. Cell invasion ability of Hep3B **(F)** and HepG2 **(G)** intervened with 0 μmol **(A)**, 20 μmol **(B)**, 40 μmol **(C)**, 80 μmol **(D)** of TanIIA. Scale bars: 50 μm. **(H–I)**. Cell invasion rates of Hep3B **(H)** and HepG2 **(I)** intervened with various concentrations of TanIIA. The data are presented as mean ± SD, *p<0.05, **p<0.01 vs the control group (0 μmol).

Three prognostic model genes were predicted by network pharmacology and machine learning experiments. In order to verify the effectiveness and reliability of these genes in the treatment of HCC, a cell model experiment was conducted to verify the results. Hep3B and HepG2 cells were intervened with different concentrations of TanIIA for 24 h, and the cell survival rate was examined by CCK-8 assay. It turned out that with the increase of TanIIA concentration, the cell viabilities of both cell lines were more inhibited, which indicated the correlation with the dose (**Figure 8A**). After analyzing the experimental data, the IC_50_ values of TanIIA for Hep3B and hepG2 were respectively calculated to be 42.45 μmol and 36.71 μmol. Therefore, the three different concentrations (low, medium and high dosing) of TanIIA were determined for subsequent experiments, whose administration concentrations were 20 μmol, 40 μmol and 80 μmol, respectively.

#### 3.3.2 The induction of TanIIA on cell apoptosis

Subsequently, whether TanIIA influenced HCC cell apoptosis or not was determined via FCM. The results of apoptosis are shown in **Figures 8D, E**. The total apoptosis rate = early apoptosis value + late apoptosis value. With the increase of TanIIA concentration, the apoptosis rate gradually increased in Hep3B cells (**Figure 8B**) as well, from (5.49 ± 0.91) % in the control group to (8.4 ± 5.57) %, (16.63 ± 2.24) % and (27.72 ± 4.22) %. And in HepG2 cells (**Figure 8C**), the apoptosis rates increased from (3.84 ± 2.89) % to (12.86 ± 1.93) %, (21.69 ± 9.11) % and (25.72 ± 4.31) %, respectively. Compared with the control group, the induction effect of TanIIA on Hep3B cells at the concentration of 80 μmol was significantly different (*p*<0.01), and there was a significant difference in the induction of HepG2 cells at the concentration of 40 and 80 μmol (*p*<0.01). Interestingly, necrotic cells were also gradually increased, especially in HepG2 cells. The number of necrotic cells was more than 20% at 80 μmol. This indicated that TanIIA may induce apoptosis and necrosis in HepG2 cells at the same time.

#### 3.3.3 The prevention of TanIIA on cell invasion

As demonstrated in **Figure 8F-I**, after intervened with TanIIA, the number of invading cells was suppressed in a dose-dependent manner. Hep3B cells intervened with 80 μmol TanIIA showed remarkably inhibition in invasion (**Figure 8H**, *p*<0.01). Similarly, it found that the invasion ability of HepG2 cells was weakened after treatment with 80 μmol TanIIA (**Figure 8I**, *p*<0.05). These results confirmed that Tan IIA can inhibit cell invasion of HCC.

#### 3.3.4 The influence of TanIIA on expression levels of apoptotic and invasion proteins

**Figure 9:**
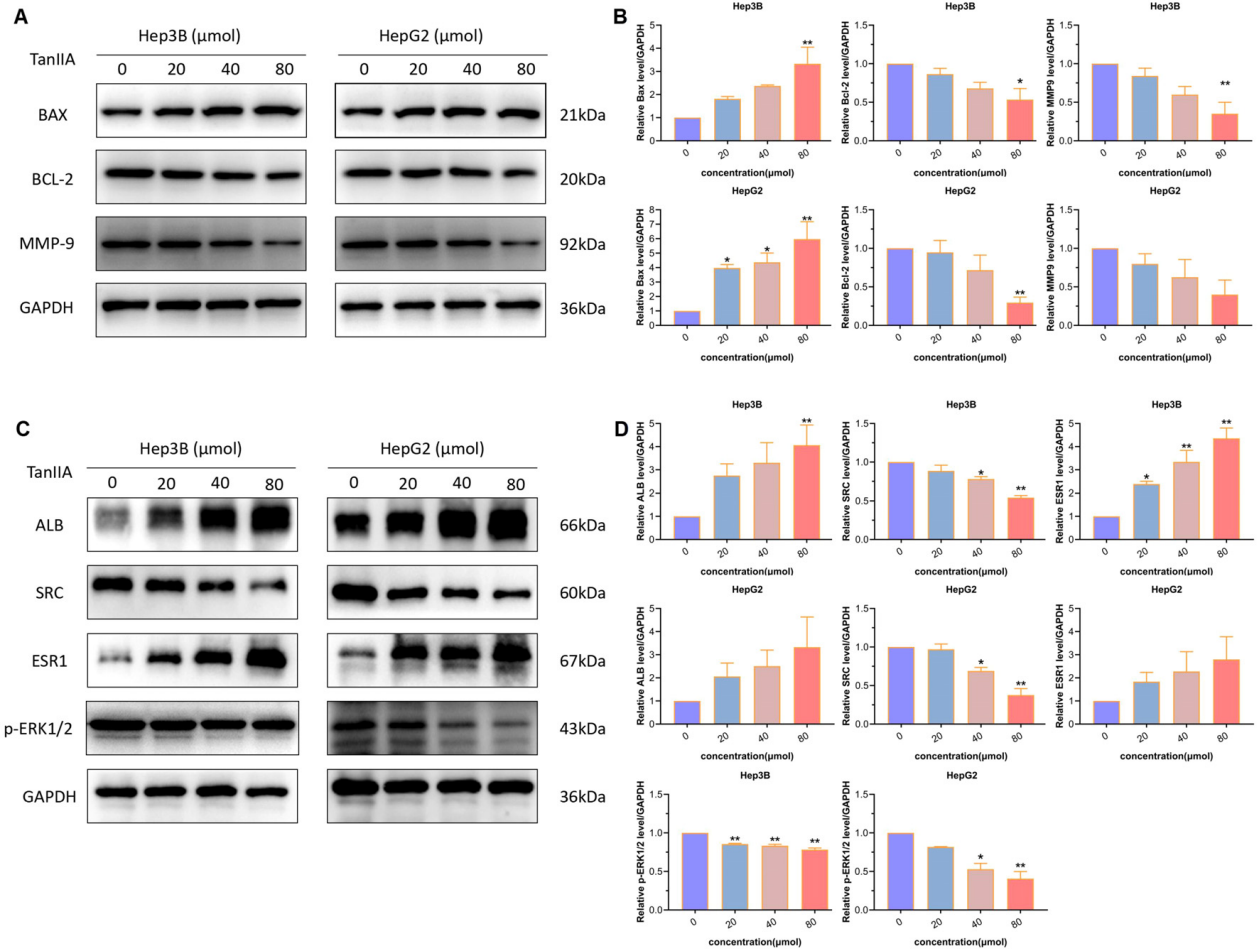
Western blotting assay. **(A, B)** Protein expression levels of apoptosis and invasion markers. **(A)**. Protein bands. **(B)**. Quantitative statistical results. **(C, D)** Protein expression levels of core genes. **(C)** Protein bands. **(D)** Quantitative statistical results. The data are presented as mean ± SD, *p<0.05, **p<0.01 vs the control group (0 μmol).

Based on the above results of FCM and transwell assay, the effect of TanIIA on HCC cells was further investigated. Therefore, the expression level changes of apoptotic proteins (Bax, Bcl-2) and invasion protein (MMP9) were detected. As shown in the **Figure 9A**, the expression of Bax showed an increase in both Hep3B and HepG2 cells intervened with TanIIA for 24h. Whilst, the expression levels of Bcl-2 and MMP9 both decreased as well. Compared with the control group, the expression levels of TanIIA-intervened group were changed with statistically significant difference (**Figure 9B**, *p*<0.05).

Taken together, these results prove that TanIIA contributes to inhibiting cell proliferation, inducing apoptosis and preventing cell invasion in HCC. It might play an important role in the anticancer progression of HCC.

#### 3.3.5 The influence of TanIIA on expression levels of core genes

Next, western blot analysis was subjected to detect the three core genes. Results indicated that the two genes, ALB and ESR1, were evoked by TanIIA (**Figures 9C, D**). And the expression levels of SRC and p-ERK1/2 were down-regulated (**Figures 9C, D**). These statistically significant results suggest that TanIIA simultaneously influences the prognostic TanIIA-related genes in HCC cells.

## 4 Discussion

TanIIA is a lipophilic active ingredient with the highest scores in Salvia miltiorrhiza, which can participate in the treatment of a variety of diseases (26, 27), especially exhibiting its excellent anti-tumor activity (28, 29). In this study, the related targets of TanIIA were predicted on the basis of multiple databases via network pharmacology. This method can predict the targets of components with more diversification and comprehensiveness, which avoids the singleness and limitation of one database.

A total of 196 potential therapeutic targets were collected by virtue of various databases, indicating that TanIIA can treat a quantity of diseases by affecting multiple targets. In order to identify the potential targets of TanIIA in the HCC treatment furthermore, we constructed a PPI network, giving a combination to the common genes of TanIIA-targeted and HCC-related genes. Then abundant TanIIA-related genes with possible therapeutic effects on HCC were screened out through the network, such as ALB, JUN, MYC, SRC, ESR1, MMP9, PTGS2 and FOS, etc.

To reveal the molecular mechanism of TanIIA in the treatment of HCC in an even better angle, we performed KEGG enrichment analysis in this study. It results displayed that the therapeutic effect of TanIIA on HCC may be achieved through MAPK, Transcriptional misregulation, Hepatitis B, TNF, endocrine resistance and other signaling pathways. MAPKs have been identified to be involved in a variety of biological and physiological processes in cells (30, 31). It is highly implicated in different cancer development processes (32). Previous studies have shown that TanIIA can induce apoptosis by activating the MAPK pathway and regulating the expression of related proteins (33, 34). Transcription genes activated via MAPK pathway consist of c-MYC, c-FOS and c-JUN, which can drive the growth and differentiation of HCC. Moreover, TNF is capable of activating growth signals by means of cytokines, consequently affecting liver cancer invasion. TNF-α and MAPK pathways can promote the proliferation and metastasis of HCC (35, 36). Extrapolating from the results of GO enrichment analysis, the regulation of TanIIA on HCC is not limited to MAPK, TNF signaling pathways, oxidative stress, hormone metabolism, inflammatory immunity, ERK1 and ERK2 cascade and other aspects. Reactive oxygen species (ROS) are closely related to tumors (37). The increase of ROS in tumors can cause a variety of biological effects, including apoptosis, autophagy, pyroptosis and ferroptosis, etc (38). Numerous studies have shown that MAPK signaling pathway plays a crucial role in various physiological processes and oxidative stress reaction (39, 40). ROS can induce MAPK signaling pathway, which can activate caspase-dependent apoptosis (41). ERK, JNK and P38 are three members of the MAPK family, in which the classical ERK1/2 is activated by growth factors and considered to be a key part in tumor progression (42). Based on the available research, TanIIA have a particular ability to reduce mitochondrial potential in tumor cells, causing the increase of mitochondrial ROS production and activation of the caspase-9 mitochondrial apoptosis pathway (43). In addition, adjuvant application of TanIIA promoted the crosstalk between ROS and p53, which can also promote cell apoptosis (44). Several studies have confirmed that TanIIA can induce Nrf2 inactivation during oxidative stress-induced apoptosis (45, 46). However, few studies pay attention to the mechanism of HCC affected by TanIIA via regulating oxidative stress. Meanwhile, the effects of TanIIA on MAPK pathway are rarely studied. This study is about to provide several theoretical possibilities for further research. According to our *in vitro* experiments, TanIIA has significantly inhibited the proliferation, induced apoptosis and reduced invasion of Hep3B and HepG2 cells in a dose-dependent manner. It might obtain some unparalleled importance on in the anti-tumor progression of HCC.

In machine learning, Lasso-penalized Cox regression acts as a useful method in determining the most important factors and improving the prediction accuracy of statistical models. Lasso is a popular machine learning algorithm that is widely used in medical research (47, 48). To further investigate the prognostic role of TanIIA treatment in patients with HCC, we used a machine learning-based Lasso regression algorithm along with cross-validation to construct a prognostic model in the TCGA database. Consequently, the model showed an excellent performance in predicting 1-year, 3-year and 5-year survival, which demonstrated a good prognostic value of this prognostic model. Additionally, we could also identify the patients in the high-risk group based on the 5-gene (ALB, JUN, SRC, ESR1 and MMP9) model. 3 core genes were picked out based on the correlation between 5 genes: ALB, ESR1 and SRC. Sorafenib is currently the only first-line therapeutic option for HCC. However, the efficacy of Sorafenib is compromised over time due to the development of drug resistance in tumor cells when used as a single-agent therapy (49). In this study, the expression of 3 core genes can influence the sensitivity of sorafenib and the immune activity of tumor micro-environments.

Among the core genes screened above, ALB is only produced by the liver, which is a marker of late maturation of liver (50), and its expression is confirmed by histopathology to reflect the function of differentiated hepatocytes (51). Previous studies suggested that the volume of HCC tumor is negatively correlated with the level of ALB. And the increased levels of ALB can inhibit the progression of HCC (52). A recent study found that ALB can interact with uPAR, accordingly inhibiting the invasion and metastasis of HCC induced by MMPs (53). What’s said above come to a suggestion that ALB is a potential tumor suppressor gene. ESR1 has been identified as a tumor suppressor gene that can predict tumor progression (54). The results of genome-wide expression analysis confirmed that ESR1 was negatively correlated with tumor size and disease stage in HCC, suggesting that the loss of ESR1 could accelerate the development of cancer (55, 56). ER-a is a protein product encoded by ESR1. Its co-localization with the transcription factor β-catenin is oriented along the Wnt pathway, which results in decreased transcription of the target genes cyclinD1 and c-myc, thereby inhibiting tumor growth in HCC. This provides a new mechanism for HCC in women by reducing the occurrence and progression (55). Accordingly, TanIIA was found to inhibit the translocation of β-catenin into the nucleus, resulting in reduced nuclear accumulation of β-catenin, which in turn reduced the expression of c-myc and cyclinD1 (57). On the contrary, SRC has a probable chance to promote the progression of HCC by positively regulating Wnt/β-catenin pathway (58). In addition, SRC also gives rise to significant activations of the MAPK/ERK pathway, leading to a series of biological effects. Highly expressed SRC takes vital association with a poor prognosis of HCC patients (59). It can be inferred that these proteins may regulate the development of HCC through the SRC/MAPK/ERK signaling axis. In this study, TanIIA was molecularly docked with the three core genes. It was turned out that the interactions with TanIIA and core genes demonstrated efficient binding. These results indicate that TanIIA can activate or inhibit the related pathways of core genes to achieve the therapeutic effect on HCC. Moreover, the changes of these three genes’ expression were evaluated. After TanIIA treatment, the expression levels of ALB and ESR1 were up-regulated, and the expression levels of SRC and p-ERK1/2 were down-regulated in Hep3B and HepG2 cells. These results are consistent with the characteristics of the genes, suggesting that TanIIA may play a therapeutic role in HCC by regulating their expression levels, and SRC/MAPK/ERK signaling pathway may be related to TanIIA in the treatment of HCC.

## 5 Conclusion

With above taken into consideration, TanIIA can suppress the growth and invasion of HCC cells, and promote the apoptosis of them. Furthermore, our findings are in favor of using TanIIA as an anti-tumor drug on account of its ability to regulate the expression of Bax, Bcl-2 and MMP9. Additionally, it acts as an anti-tumor agent by inhibiting SRC/MAPK/ERK pathways and regulating the expression of ALB, ESR1 and SRC. And these three genes proved to be reliable prognostic molecules in our experiments. Mechanistically, results mentioned above tend to demonstrate a new targeted treatment strategy for HCC with significant therapeutic effect.

## Data Availability

The original contributions presented in the study are included in the article/**Supplementary Material**. Further inquiries can be directed to the corresponding authors.
