# Supplementary material for: In silico identification and verification of Tanshinone IIA-related prognostic genes in hepatocellular carcinoma
Source: Front Immunol. 2024 Oct 31;15:1482914. doi: 10.3389/fimmu.2024.1482914 (PMC11560438; doi:10.3389/fimmu.2024.1482914)
Supplement: Supplementary file 1 [file Table1.docx]

Table S1 pharmacological and molecular properties

| MW | AlogP | Hdon | Hacc | OB (%) | Caco-2 | BBB | DL | RBN |
| --- | --- | --- | --- | --- | --- | --- | --- | --- |
| 294.37 | 4.66 | 0 | 3 | 49.89 | 1.05 | 0.70 | 0.40 | 0 |
